# An Update on mRNA-Based Viral Vaccines

**DOI:** 10.3390/vaccines9090965

**Published:** 2021-08-29

**Authors:** Subbiah Jeeva, Ki-Hye Kim, Chong Hyun Shin, Bao-Zhong Wang, Sang-Moo Kang

**Affiliations:** Center for Inflammation, Immunity & Infection, Institute for Biomedical Sciences, Georgia State University, Atlanta, GA 30303, USA; jsubbiah@gsu.edu (S.J.); kkim39@gsu.edu (K.-H.K.); cshin@gsu.edu (C.H.S.); bwang23@gsu.edu (B.-Z.W.)

**Keywords:** mRNA vaccines, SARS-CoV-2, influenza

## Abstract

With the success of COVID-19 vaccines, newly created mRNA vaccines against other infectious diseases are beginning to emerge. Here, we review the structural elements required for designing mRNA vaccine constructs for effective in vitro synthetic transcription reactions. The unprecedently speedy development of mRNA vaccines against severe acute respiratory syndrome coronavirus 2 (SARS-CoV-2) was enabled with previous innovations in nucleoside modifications during in vitro transcription and lipid nanoparticle delivery materials of mRNA. Recent updates are briefly described in the status of mRNA vaccines against SARS-CoV-2, influenza virus, and other viral pathogens. Unique features of mRNA vaccine platforms and future perspectives are discussed.

## 1. Introduction

Severe acute respiratory syndrome coronavirus 2 (SARS-CoV-2) was identified as the causative agent for the rapidly transmitting pandemic coronavirus disease 2019 (COVID-19) across the globe since the first outbreak in December 2019 [1,2]. With a strong consensus, there were unprecedented efforts and global coordination in developing COVID-19 vaccines to control the pandemic sustainably. The vaccine platforms in a developmental race included conventional approaches such as adjuvanted whole inactivated virus, subunits, and viral vectors, as well as genetic vaccines resulting in a renaissance of RNA vaccines despite unproven mRNA vaccine technologies on the market [3,4]. Just 64 days after the sequence information of novel SARS-CoV-2 RNA was available [1], the first USA clinical trial started with volunteers receiving the Moderna mRNA-1273 vaccine candidate [5,6,7]. With promising early safety and efficacy results from Moderna (mRNA-1273) and Pfizer/BioNTech mRNA (BNT162b2) vaccine candidates, phase 3 trials were initiated on 27 July 2020 [8]. As a consequence of successful completion of phase 3 clinical studies, these two mRNA-based SARS-CoV-2 vaccines were proven to be safe and 94 to 95% efficacious in both healthy adults and elderly populations [9,10,11].

It is a miraculous scientific triumph to develop novel mRNA vaccines, licensed first in the United Kingdom and then Canada and the USA, for emergency use authorization for humans in an actual pandemic situation within a year with an unprecedented shorter time and higher efficacy than traditional vaccine platforms. COVID-19 mRNA vaccines are not the whole story of the RNA vaccine field. Scientists and companies had been developing, innovating, and improving RNA technologies for almost three decades prior to COVID-19. In the 1990s, the injection of mRNA into mouse muscle resulted in protein expression [12], and antigen-specific T cell responses were induced by immunization with mRNA encoding influenza virus nucleoprotein [13]. However, inflammation and toxicity associated with unmodified mRNA and delivery vehicles were recognized as challenging problems in translating mRNA vaccines to humans since RNA itself can be a reactogenic inflammatory molecule. In 2005, the inclusion of modified nucleosides in mRNA transcripts produced significantly lower reactogenic and inflammatory responses [14], improving the vaccine safety and enhanced mRNA translation efficacy in later studies [15,16,17,18]. Naked mRNA is ineffective in entering the cells, unstable, and easily destroyed. The development of lipid nanoparticles (LNP) to facilitate the delivery of RNA molecules into the cells in vivo has become a major step in innovating RNA technologies [19,20]. The first clinical phase 1 studies using modified mRNA vaccines in LNP were against influenza virus H10 and H7 hemagglutinin (HA) during the years between 2015 and 2018, resulting in 100% seroconversion [21,22]. The success of COVID-19 mRNA vaccines has proven that RNA technology, as a new platform, is safe and effective for commercial production.

Here, we review the structural elements in designing mRNA vaccine constructs, the parameters to be optimized, the current updates of mRNA-based vaccines against viral diseases, and the unique features of mRNA vaccine technology in an era of a pandemic. New perspectives and future applications of mRNA technology are discussed to address the development of novel mRNA vaccines against challenging and recurring viruses and the antigenic variants of viral pathogens.

## 2. Structural Elements in Constructing DNA Template for In Vitro mRNA Synthesis

**Figure 1:**
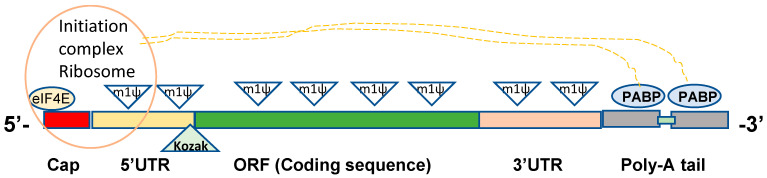
Structural elements for effective in vitro transcription of mRNA. 5′Cap: 7-methylguanosine (m7GpppN) or additional methylation (Cap1: m7GpppN^m^pN) required for recognition by eukaryotic translation initiation factor 4E (eIF4E); 5′UTR and 3′UTR: 5′ and 3′ untranslated regions required for ribosome binding and translation initiation complex formation; ORF: open reading frame encoding a gene of interest; poly-A tail: recognition by poly-A binding protein (PABP) to initiate translation, forming a hypothetical loop structure with initiation ribosome complex; m1ψ: N1-methylpseudouridine instead of uridines; Kozak: the Kozak sequence.

For efficient translation, as detailed in previous review articles [23,24], in vitro transcribed (IVT) mRNA products should have critical structural elements, which include the 5′ cap, untranslated regions (UTR) on both ends, open reading frames (ORF) encoding proteins, and a poly-A tail (Figure 1). The 5′ cap at the 5′ end of the mRNA strand is required for protection from exonuclease attack, recognition by eukaryotic translation initiation factor 4E (eIF4E), and to promote the translation initiation complex of ribosomes [25,26]. The UTRs of mRNAs contribute to stability, translation efficiency, and recruiting mRNAs to the ribosomes [27,28]. ORF in IVT mRNA contains coding sequences to be translated into proteins. Current COVID-19 mRNA vaccines are in vitro manufactured, replacing uridine bases with N1-methylpseudouridine (m1ψ) to improve safety and translation efficacy (Figure 1) [23,29,30,31,32]. The poly-A tail also contributes to mRNA stability and is required for recognition by poly-A binding protein (PABP), which in return interacts with ribosome initiation complex for effective translation [33,34,35]. The Pfizer/BioNTech mRNA vaccines were designed to contain two segmented poly-A tails with a short spacer [23]. Essential roles of IVT mRNA structural elements are detailed in the following sections.

### 2.1. 5’ Cap

The 5′ capping in mature mRNA is required for protection of mRNA from degradation, facilitating recruitment of the ribosomes, gene expression, and self- versus non-self-identification. Several variations of 5′ cap structures have been found to exist in nature [36,37]. The 5′ cap of the eukaryotic mRNA contains 7-methylguanosine (m7G) through a 5′-5′-triphosphate bridge (m7GpppN) via a series of enzymatic capping reactions involving RNA triphosphatase, guanosyltransferase, and S-adenosyl methionine [38]. To further enhance the translation efficiency, additional methylation was introduced at the first nucleotide (cap1: m7GpppN^m^pN) or both first and second nucleotides (cap2: m7GpppN^m^pN^m^) [24]. Another simple approach is to co- transcribe into RNA with cap analogs (e.g., m7GpppG) at an excess amount, but in this case a certain level of uncapped mRNA and a reverse orientation of cap (Gpppm7G) can be generated [39]. To avoid this drawback, enhanced translation efficacy was reported with an anti-reverse cap analog (ARCA: 3′-O-Me-m7GpppG) where the 3′-OH hydrophilic group was methylated to block reverse incorporation by RNA polymerase [40]. A new strategy of co-transcription was to specifically add a natural 5′ cap1 structure to the start site during IVT reactions using a CleanCap kit (TriLink Biotechnology), simplifying the 5′ capped mRNA production by T7 polymerase in vitro reactions [37,41], which has become a commonly used capping method resulting in high translation and low reactogenicity. An approach of CleanCap was used for 5′ capping of COVID-19 BNT162b1 mRNA vaccines [42].

### 2.2. 5′ UTRs

The major role of mRNA UTRs is to control post-transcriptional gene expression. The 5′ UTR is critical for ribosome recruitment and represents the site forming the preinitiation complex of protein translation (Figure 1) [43]. The 5′ UTR sequence and secondary structures affect the mRNA stability and translation efficacy as proposed by a scanning model of RNA translation [43,44,45]. The design of mRNA UTRs influences the level of proteins expressed, which should avoid the formation of stable hairpin structures and consider an appropriate distance (1–46 bases) to the 5′ G cap and GC content in the 5′ UTR [44]. Alpha-globulin, heat-shock protein, or viral UTRs were used in earlier studies to enhance translation efficiency [16,24]. Systematic optimization of mRNA 5′ UTR based on the variables such as 5′ UTR length and Kozak sequence was reported to enhance expression of therapeutic mRNA products, revealing the critical aspects and roles of UTRs in improving and engineering DNA templates for IVT mRNA synthesis [27]. Effective translation via the optimized 5′ UTR constructs was more critical for mRNA expression than mRNA stability, suggesting the essential roles of 5′ UTR for protein production. Throughout the combinatorial library screening, the 5’ UTR sequences derived from complement factor 3 and cytochrome p4502E1 were reported to enhance protein expression regardless of the 3′ UTR [27].

The specific motif and internal ribosomal entry site (IRES) can control ribosome binding to enable cap-independent mRNA universal translation even in the cells with eIF4E at a low level, but cap structure is still needed to protect from exonuclease attack [46,47]. IRES of various viruses (encephalomyocarditis virus, tobacco etch virus) is often included in the 5′UTR [48]. After ribosome binding, the initiation complex recognizes the start codon (AUG) in the Kozak sequence of mRNA and initiates the translation process [27,43,44]. The six nucleotides known as the Kozak sequence at the junction of the 5′ UTR and the open reading frame with the start AUG codon greatly impact the efficiency of protein translation (Figure 1), indicating the importance of an optimized Kozak sequence [45,49].

### 2.3. 3′ UTRs

While the 5′ UTR promotes the initiation of translation, specific sequences in the 3′UTR contribute to stabilizing intracellular mRNA, ultimately enhancing the duration of protein expression [50]. Longer 3′ UTRs are known to have a shorter half-life of mRNA whereas shorter 3′ UTRs are known to be less effective in translation of mRNA, suggesting an optimal length requirement of 3′ UTR mRNA [51,52]. The 3′ UTR of α-globin mRNA contains pyrimidine-rich motifs, forming a messenger ribonucleoprotein α-complex and stabilizing the poly-A binding protein to the poly-A tail, and providing exceptional mRNA stability [53]. Globin UTRs are commonly included in the design of in vitro synthetic mRNA to enhance mRNA stability and performance [24,53]. The 3′ UTR also contributed to high expression by retaining the elongation factor 1 A1 [54]. These UTR sequences were shown to increase IVT mRNA stability and expression in several cell lines [52,55]. A recent study using a cellular library and cell culture screening reported new dual UTRs of combining mitochondrially encoded 12S rRNA and amino-terminal enhancer of split mRNA, resulting in comparable or superior to the broadly used human β-globin 3′ UTR for protein expression of IVT mRNA [41,51].

### 2.4. ORFs

An ORF of mRNA dictates the primary sequence information of the target protein of interest and higher order RNA structures impacting translation efficacy. Codon usage and RNA secondary structures independently contribute to regulating protein expression, suggesting the difficulty of optimization. Unexpectedly, mRNA coding sequences potentially forming secondary structures were shown to be correlated with highly expressed mRNAs [56]. In addition, modified bases (m1Ψ, Ψ, methoxyuridine) moderately stabilizing mRNA secondary structures were reported to enable high expression of a wide variety of mRNAs with different primary sequences [56]. This is in contrast to suppression of translating mRNA with structured 5′ UTRs [45] and particularly with modified bases [57]. Further studies suggested that mRNA secondary structures that can be stabilized further by m1Ψ may increase the functional half-life of mRNA independent of codon optimality [56,58]. IVT mRNA stability and protein expression were increased by reducing the frequency of UU and UA dinucleotides, probably due to the protection from endonuclease [59] or by depleting uridines via chemical modification [60]. An alternative approach to reduce inflammatory responses to mRNA was to limit the uridine usage in the codons by engineering mRNA sequence [61]. Additionally, optimization of codon usage in synthetic mRNAs mediates translation efficiency through preventing premature termination at rare codons [62,63]. Simply optimizing the codon usage would not work for maximizing mRNA translation efficacy. Multiple variables should be considered in designing ORFs for IVT mRNA, which include putative secondary mRNA structures, codon usage, and mRNA stability in vivo [64,65,66].

### 2.5. Poly-A Tail

The poly-A tail, which is a typical length of 60 to 150 nucleotides, is essential for mRNA stability, translation, and recognition by poly-A binding protein (PABP) that subsequently interacts with the translation initiation complex (eIF4G) to form a loop-like conformation (Figure 1) [33]. Two methods are commonly used to produce poly-A-tailed IVT mRNA. One is an enzymatic polyadenylation approach using recombinant poly-A polymerase adding poly-A tail to the 3′end of IVT mRNA after synthesizing mRNAs, which produces a different length of poly-A and less consistent batch controls, which will make it difficult to meet regulatory requirements [67]. Another method is the co-transcription of poly-A tail during IVT mRNA synthesis using a DNA template with poly-T nucleotides, generating homologous mRNA products [68]. A plasmid template-encoded poly-A tail is prone to recombine and to shorten its poly-A tail length [69]. An approach of using segmented poly-A repeats was reported to reduce recombination of plasmids during production by including 40–60 adenosines that are a sufficient length for binding to PABP and separated by a spacer element [69]. The mRNAs with segmented poly-A tails have the superior potential of high mRNA performance of half-life and translation efficiency compared to homologous poly-A tails [69]. The Pfizer/BioNTech mRNA vaccine has a strategy of utilizing two segmented poly-A tails [23].

## 3. In Vitro Transcription and Modification of Synthetic mRNA

Once the DNA template is designed and constructed to contain a promoter, UTR, ORF, and poly-T sequences as described above (Figure 1), mRNA can be synthesized via in vitro transcription using recombinant T7 RNA polymerase, an extraordinarily capable enzyme with several unique features [23,70,71]. T7 RNA polymerase can generate longer RNAs than 20,000 nucleotides free of errors and incorporate pseudouridine triphosphate and other modified nucleotides without altering base-pairs [72,73,74]. Chemical modifications of mRNAs were shown to improve protein expression, dependent on the cell types and coding sequences [72]. The Pfizer/BioNTech BNT162b2 mRNA contains N1-methylpseudouridine (m1ψ) instead of every uridine residue in the coding sequence and UTRs recognized by the ribosome (Figure 1), indicating that modified nucleobases are compatible with all of its mRNA functional elements [23].

For co-transcription of capping, IVT reaction solution is prepared to contain cap analogs in optimized ribonucleoside triphosphate substrates, RNA polymerase, and DNA template. Similar to viral RNAs, the host immune system recognizes IVT unmodified mRNAs as exogenous danger signals, activating pattern recognition receptors such as Toll-like receptors (TLRs) and cytoplasmic RNA sensors. Endosomal TLR7 and TLR8 recognize single-strand RNA as ligands, while TLR3 binds to double-strand RNA molecules [75,76,77]. The cytoplasmic RNA sensors include RNA-dependent protein kinase R (PKR) [15], retinoic acid-inducible gene-1 (RIG-1), and melanoma differentiation-associated protein 5, recognizing double-stranded and 5′-triphosphate-modified RNA [78,79,80]. Induction of interferons and activation of PKR via RNA stimulation resulted in phosphorylation of eIF2a, inhibiting mRNA translation and protein expression [15,81]. Uncontrolled systemic induction of excess inflammatory cytokines can lead to safety concerns of allergic reactions and anaphylactic shock, rare fatal outcomes that prevented the development of previous mRNA therapeutics to clinical application [82,83]. To overcome this issue of activating the innate immune system via recognition of mRNA, modified nucleosides incorporated into the IVT mRNA were found to significantly suppress the innate immune responses and to enhance the mRNA translation efficacy and protein expression [14,15,16,17,18]. Naturally occurring pseudouridine (ψ) is commonly introduced into IVT modified mRNA during synthesis. N1-methylpseudouridine (m1ψ) or Ψ-incorporated mRNA is non-reactogenic or significantly less reactogenic and does not stimulate the innate immune system but increases mRNA stability and translation capacity of protein expression [15,16,18,84]. A later study reported that m1ψ outperforms ψ in driving high levels of protein production and evading TLR3 activation [85]. Other modified nucleosides such as 5-methoxyuridine were also reported with improving mRNA stability and protein expression [72].

There are additional studies demonstrating the mechanisms of lowering stimulation of the innate immune system in base-modified IVT mRNA products. Use of base-modified nucleotides led to reduced synthesis of antisense duplex mRNA by-products and yielded less inflammatory mRNA [86,87]. Secondary structures of mRNAs containing m1Ψ differ from mRNA with uridine, resulting in less stimulatory immune signaling through RIG-1 [56,88]. Incorporation of m1Ψ in RNA altered TLR7 recognition, and thus m1Ψ mRNAs were less stimulatory in expressing inflammation genes than those with mRNAs with uridine [23,89]. Based on the observation that incorporation of m1Ψ into mRNA was shown to increase the size and abundance of ribosomes, it was proposed that the more rapid translation initiation and slower elongation of m1Ψ mRNAs might coordinately increase productive interactions with the poly-ribosome, providing evidence of direct impact of m1Ψ mRNA translation [25]. It is important to note that modified mRNA does not work in all protein production. Erythropoietin production was higher by unmodified mRNA containing viral IRES in 5′ UTR than Ψ-modified mRNA [61].

## 4. Lipid Nanoparticle Delivery of Synthetic mRNAs

IVT mRNA transcripts are unstable and highly susceptible to degradation by nucleases as evidenced by a short half-life (<5 min) in sera [90]. Most cells resist taking naked mRNA intracellularly through lipid bilayer membranes [91]. A significant innovation is the development of nanomaterials protecting mRNA from degradation and enabling the delivery of mRNA to the cytoplasm without toxicity. Lipid nanoparticles were formulated to encapsulate mRNA transcripts in recent COVID-19 mRNA vaccines licensed on the market (Table 1) (reviewed in [92]). The mRNA nanoparticles are prepared through a self-assembly process at low pH by rapidly mixing lipid formulations and mRNA vaccines [20]. The efficacy of delivering mRNAs and immune responses is comparable to a viral vector containing the same gene [93]. The components of LNP typically include an amine-group ionizable lipid, cholesterol, PEGylated lipid, and a helper lipid such as distearoyl- phosphatidylcholine (DSPC) [94]. The ionizable amino lipid plays a critical role in functioning mRNA LNP as biodegradable lipids were identified and incorporated into formulating nanoparticles [20]. Improved biodegradability of ionizable lipids reduced inflammation in the injection site, resulting in more acceptable tolerability and minimum exposure to other tissues due to rapid metabolic breakdown and clearance.

## 5. Immunogenicity of mRNA Vaccines

The mRNA vaccines after intramuscular injection are taken up by antigen-presenting cells via endocytic pathways [95]. The mRNA in the cytoplasm can activate innate sensors and is translated into expressing immunogenic proteins intracellularly, mimicking viral infection and inducing potent T cell and B cell responses. The FDA-approved SARS-CoV-2 mRNA vaccines (mRNA-1273, BNT162b2) were reported to exhibit high efficacy of over 94% in phase 2 and 3 studies [9,96]. Since extensive preclinical and clinical data are available for SARS-CoV-2 mRNA vaccines, we briefly summarize immune responses correlating with the high efficacy of mRNA vaccines tested against SARS-CoV-2 as a representative. Previous studies on influenza virus HA mRNA vaccines demonstrated the induction of T follicular helper cells after vaccination of non-human primates [97,98]. Immunization of mice with SARS-CoV-2 mRNA vaccines induced significantly increased germinal center reactions and T follicular helper cells in draining lymph nodes and spleens [99,100,101]. In addition, SARS-CoV-2 mRNA immunization induced effector CD4 T cells secreting T helper type 1 cytokines (IFN-γ, TNF, IL-2) and CD8 T cells with IFN-γ and IL-2 production [30,99]. Clinical mRNA lipid nanoparticle vaccines that encode a full-length, prefusion stabilized spike (S) protein induced S-specific IgG antibodies capable of neutralizing pseudovirus with spike and wild type SARS-CoV-2 in mice even after a single dose ranging from 0.2 to 10 µg mRNA [30,99,102,103]. High single-dose immunization of mice with mRNA (15 µg) encoding the spike receptor-binding domain (RBD) [104] or mRNA of the full-length spike with cleavage mutation (30 µg) could induce sustained neutralizing antibody responses, germinal center formation [100], long-lived plasma and memory B cells, and type 1 CD4 T and CD8 T cell responses [30]. With a prime-boost strategy, low dose ranges (1 or 2 µg) of mRNA vaccines encoding a stabilized prefusion spike could induce neutralizing antibodies in mice [30,105,106]. A full-length S mRNA vaccine appeared to be more effective in inducing neutralizing antibodies than the S1 subunit mRNA vaccine, suggesting the importance of an appropriate choice of immunogens [101]. Overall, SARS-CoV-2 mRNA vaccines were highly immunogenic in inducing S-specific IgG and neutralizing antibodies as well as CD4 and CD8 T cell immune responses, correlating with the in vivo protection.

## 6. SARS-CoV-2 Synthetic mRNA Vaccines

Research developments and innovations in the use of synthetic mRNA methods and processes for several decades led to the first Phase 1 clinical studies on influenza hemagglutinin (HA) mRNA lipid nanoparticle vaccine in 2015 [21,22]. A previous study on the pre-fusion stabilized structure of Middle East respiratory syndrome coronavirus (MERS-CoV) spike (S) protein paved the way for the rapid development of the Moderna pre-fusion S mRNA-1273 vaccine [30,107,108]. Pfizer/BioNTech BNT162b2 mRNA encodes full-length prefusion S with a furin cleavage site deleted, which was shown to be more immunogenic in mice compared to its wild type counterpart [32]. Moderna mRNA-1273 vaccine expresses prefusion S protein via two consecutive proline substitutions (2P; K986P and V987P) and retains an intact S1–S2 cleavage site [7,30]. TranslateBio mRNA S prefusion vaccine (MRT55500) was stabilized by dual mutations of 2P and the cleavage site (GSAS from RRAR polybasic residues), which was selected from comparison of wild type S and multiple S mutants including 2P, GSAS, 2P/GSAS, 6P, and 6P/GSAS in mice and non-human primates [109]. Novavax SARS-CoV-2 S nanoparticle protein vaccine candidate (NVX-CoV2373) is a full-length spike with similar double mutant 2P–3Q (QQAQ from RRAR cleavage site) [110]. Substitutions of 2P and 3Q might have contributed to stabilizing prefusion S conformation and inducing protective immunity but their effects and safety are not fully understood yet in human vaccination.

With unprecedented speedy preparedness and effort, clinical phase 1 and 2 studies and phase 3 efficacy trials of mRNA vaccines were initiated and carried out approximately 2 and 4 months after sequence availability in the real COVID-19 pandemic situation [111,112]. The mRNA-1273 and BNT162b2 COVID-19 vaccines from Moderna and Pfizer/BioNTech, respectively, first approved for emergency use authorization, are conventional base-modified (m1ψ) non-replicating mRNA vaccines encoding prefusion stabilized S proteins with transmembrane (TM) domain formulated in LNP (Table 1). Pfizer/BioNTech BNT162b1 mRNA vaccine encoding SARS-CoV-2 RBD was tested in phase 1 and 2 trials, reporting comparable neutralizing antibodies but with a higher incidence of systemic reactions than the BNT162b2 full-length S mRNA vaccine [113,114,115]. Other ongoing clinical tests of COVID-19 vaccines include unmodified mRNA prefusion S, CVnCoV (from CureVac), and self-amplifying mRNA prefusion S ARCT-021 (from Arcturus) at a range of lower doses (Table 1). TranslateBio/Sanofi and Imperial College London are also developing unmodified mRNA and self-amplifying mRNA vaccines, respectively. The phase 2b/3 clinical study on CureVac unmodified mRNA vaccine recently reported disappointing late-stage results (approximately 47% efficacy) for CVnCoV [116].

Self-amplifying mRNA contains cis or trans RNA transcripts encoding non-structural proteins forming RNA-dependent RNA polymerase (RDRP), as well as mRNA for protein antigens of interest [112]. Owing to the nature of RDRP to amplify mRNAs encoding for antigens of interest, possible dose sparing effects were reported in self-amplifying mRNA lipid nanoparticle strategies (Table 1). Challenges exist in unmodified mRNA and self-amplifying mRNA approaches, including nuclease degradation, stimulation of innate immunity, and protein expression. The bigger size of self-amplifying mRNA constructs to include RDRP- and protein antigen-encoding mRNA are expected to be more complex and challenging in scaling up production, stability, and purification.

**Table 1:** SARS-CoV-2 mRNA vaccines in lipid nanoparticle formulations.

Company /Sponsor	mRNA Type	Immunogens	mRNA Dose (μg)	Clinic Data	Preclinical Data
Moderna	Base-modified mRNA	mRNA-1273: prefusion stabilized Spike-TM	100	FDA approved [9]	[30,31]
Pfizer/BioNTech	Base-modified mRNA	BNT162b2: prefusion stabilized Spike-TM	30	FDA approved [10]	[32]
Pfizer/BioNTech	Base-modified mRNA	BNT162b1: receptor binding domain	30	phase 2 [114]	
CureVac	Unmodified mRNA	CVnCoV: prefusion stabilized Spike-TM	12	phase 2b/3 [116]	[105]
Arcturus	Self-amplifying mRNA	ARCT-021: full length Spike	1–10	phase ½	[117]
Imperial College	Self-amplifying mRNA	full length Spike	1–10		[118]
TranslateBio /Sanofi	Unmodified mRNA	prefusion stabilized Spike-TM (MRT55500)	7.5		[109]

Spike-TM: full-length spike with transmembrane domain.

## 7. Influenza mRNA Vaccines

Before COVID-19, mRNA-based influenza vaccines were investigated extensively in preclinical studies due to the ease of testing efficacy in small animal models, which led to the first clinical trials [21,22] and paved the road toward rapidly developing SARS-CoV-2 mRNA vaccines. In 2012, sequence optimized, nucleoside-unmodified HA (H1, H3, H5) mRNA vaccines formulated in protamine complexes were tested in mice, ferrets, and pigs, providing early conceptual work on protection by mRNA vaccination (Table 2) [119]. Self-amplifying mRNA formulated in cationic nanoemulsions provided protective immunity even at a low (0.1 µg mRNA) dose in mice [120]. With innovation in delivery vehicles, LNP-formulated mRNA influenza vaccines have been investigated since 2015. Nucleoside modified mRNA LNP vaccines encoding HA (H1N1, H10N8, H7N9) were tested for their immunogenicity and efficacy in different animal models. A codon optimized, nucleoside unmodified HA (H1N1 2009 pandemic) mRNA vaccine (10 µg) could induce protective hemagglutination inhibition (HAI) titers for a year in non-human primates (NHPs) even with single intramuscular immunization [121]. Injection of NHPs with modified non-replicating H10 HA mRNA (50 μg) LNP resulted in uptake and translation of mRNA in monocytes and dendritic cells, inducing CD4 T cell responses and protective HAI titers [95]. Intradermal immunization of mice with modified H1 HA (H1N1 2009 pandemic) mRNA LNP (10–30 μg) induced protective HAI and stalk antibodies, providing homologous and heterologous protection in mice [122]. T follicular helper cells and germinal centers were induced by vaccination with HA mRNA LNP, indicating the generation of quality B cell responses [97,98]. Neuraminidase (NA) mRNA LNP vaccination was shown to induce homologous protection, preventing weight loss with as low an mRNA dose as 0.5 µg [123]. A combination of multi antigenic (NA+ M2+ Stalk +NP) mRNA (20 μg) LNP vaccines was highly effective in conferring broad cross-protection in mice, suggesting a promising approach for universal influenza vaccination [123]. Before COVID-19, the first phase 1 trial studies demonstrated that H10 mRNA (100 μg) LNP and H7 mRNA (100 μg) LNP vaccines were well tolerated and elicited protective HAI titers and micro-neutralizing antibodies in healthy adults [21,22], providing pivotal cornerstone clinical data for the speedy development of mRNA-based SARS CoV-2 vaccines.

## 8. Other Viral mRNA Vaccines

The development of mRNA-based vaccines against infectious diseases has been previously summarized in self amplifying and conventional non-replicating RNA technologies [112,124,125]. This section reviews recent updates on preclinical and clinical studies on mRNA vaccines against other viruses, including human respiratory syncytial virus (RSV), Zika virus, rabies, and human immunodeficiency virus (HIV).

Nucleoside modified RSV mRNA (m1ψ-mRNA/LNPs) vaccines encoding either prefusion F stabilized or wild type fusion (F) were reported to be effective in inducing neutralizing antibodies in mice (10 μg) and cotton rats (25 μg), as well as CD4^+^ and CD8^+^ T-cell responses in mice [126]. A phase 1 study using RSV prefusion F mRNA LNP vaccine was carried out in healthy young and old adults to assess the safety and immunogenicity [127]. All dose ranges (25 to 200 μg mRNA in young adults, 25 to 300 μg mRNA in old adults) were tolerated without severe adverse events, raised RSV neutralizing titers, prefusion F-specific antibodies, and cell-mediated immune responses to RSV F peptides [127].

Zika virus (ZIKV) pre-membrane and envelope (prM-E) glycoprotein encoding modified mRNA LNP vaccine provided protection against ZIKV challenge in mice (30 μg mRNA) and in NHPs (50 μg mRNA) after a single dose [128]. Sterilizing immunity against ZIKV was also reported in mice with two doses of modified mRNA LNPs encoding prM-E genes avoiding potential cross-reactivity with the dengue virus [129]. A platform of self-amplifying mRNA encoding ZIKV prM-E protected mice with a low dose mRNA vaccine [130]. A phase 1 clinical trial study (NCT03014089) with the ZIKV mRNA LNP vaccine is ongoing [125].

A non-replicating mRNA vaccine encoding rabies virus glycoprotein (Rabies-G) could induce neutralizing antibodies that last for one year, CD4 and CD8 T cell responses, and protect against lethal intracerebral challenge in mice [131]. A thermostable Rabies-G mRNA vaccine was developed to retain immunogenicity and protective characteristics during storage at different temperatures up to 56 °C, avoiding the extreme cold chain distribution of mRNA vaccines [132]. In phase 1 study (NCT03713086), low-dose unmodified Rabies-G mRNA LNP vaccine formulation was well tolerated and induced rabies neutralizing antibodies after boost vaccination [133].

HIV-1 envelope gp160 encoding nucleoside-modified mRNA LNP vaccination induced polyfunctional HIV-1 antibodies at a similar or superior magnitude and breadth compared to adjuvanted protein immunization and durable neutralizing antibodies for 41 weeks in NHPs [134]. Nucleoside-modified mRNA LNP vaccine encoding human cytomegalovirus (HCMV) glycoprotein B (gB) elicited IgG antibodies with more outstanding durability and breadth compared to MF59-adjuvanted gB protein immunization in rabbits [135] and in mice and NHPs [136]. These studies support mRNA LNP as a viable strategy for enhancing the efficacy of HCMV vaccination. A phase 1 study (NCT03382405) using a HCMV mRNA LNP vaccine is ongoing [125].

## 9. Safety of COVID-19 Vaccines

It is advised not to administer the COVID-19 mRNA LNP vaccine in subjects with a significant history of allergic reactions because of potential severe adverse effects [137]. Polyethylene glycol (PEG) is a hydrophilic polymer that is included in COVID-19 mRNA LNP formulated vaccines and used as an excipient in daily products such as medicinal drugs, cosmetics, or foods. It remains unknown what triggered the adverse allergic reactions as reported at the time of early vaccination campaign in two healthcare workers after getting a BNT162b2 mRNA vaccination, although allergic reactions to PEG were described in individuals who developed anaphylactic responses to medications containing PEG [138]. Other side effects such as fever, sore muscles, and body aches are common in some individuals similar to other vaccinations, which are not specific to the mRNA vaccines or the spike protein.

Endogenous reverse transcriptase (RT) activity was shown to serve as a translational repressor in platelets via RNA–DNA hybrids and treatment with RT inhibitors resulted in enhanced global protein synthesis and platelet activation [139]. A recent study reported that the BNT162b2 mRNA vaccination does not alter platelet protein activation [140], suggesting that foreign RNA is unlikely to activate platelets. In contrast, vaccination with ChAdOx1 nCov-19 (AstraZeneca) may result in very rare events of producing autoantibodies against platelet-factor 4, potentially causing catastrophic thrombotic thrombocytopenia disorder in the cerebral venous [141,142]. It has been suggested that these rare events of autoantibodies and thrombocytopenia might be related to adenoviral vector vaccines (Ad26.COV2.S, ChAdOx1 nCov-19), unlike mRNA-based vaccines [143]. Meanwhile, SARS-CoV-2 infection and COVID-19 can lead to dysregulation of platelet expression, thrombocyte activation, and adverse cardiovascular events [144].

## 10. Conclusions and Perspectives on mRNA-Based Viral Vaccines

The success of mRNA-based COVID-19 vaccines has proven the unique features of mRNA vaccine technology. Numerous conventional and new vaccine platforms competed with one another to develop COVID-19 vaccines right after the sequence information of the new SARS-CoV-2 was available. The mRNA vaccines from Pfizer/BioNTech and Moderna quickly turned out to be the frontrunners, obtaining the emergency use approval for prophylactic COVID-19 vaccination within 11 months. The speed of developing mRNA-based COVID-19 vaccines was much faster than any other vaccine platform, probably owing to the nature of IVT mRNA preparations, which does not require cell culture, biohazard materials, and complex purification procedures. Of course, it is an important lesson that the quicker development of mRNA COVID-19 vaccines resulted from almost three decades of continued research efforts and innovations in mRNA technology. The efficacy (~95%) of protection by mRNA vaccination [11] is exceeding our expectations and is much higher than other platforms of licensed vaccines against respiratory pathogens. Another advantage is that mRNA vaccines do not induce vector immunity, thus not interfering with subsequent vaccinations. Additionally, pre-existing immunity would not affect mRNA vaccination because of intracellular expression of protein antigens, although its effects remain to be tested. Since the success of COVID-19 mRNA vaccines resulted in establishing a regulatory and manufacturing network and infrastructure, new RNA vaccines will not go through lengthy safety tests and production lines for clinical trials. In contrast to DNA-based vaccines, mRNAs in nature would not have the potential risk of integration into the host chromosomes, leading to mutagenesis.

Now the mRNA vaccine technology has the versatility of substantial room for fine-tuning molecular design regardless of antigens. The commercial scale-up manufacturing process would stay the same for any other mRNA sequence. The success of mRNA-based COVID-19 vaccines is opening up new avenues of other mRNA vaccines against recurring infectious diseases such as influenza, RSV, rabies, Zika, HIV, herpes, dengue, hepatitis, and malaria. The quicker production of mRNA vaccines will be more responsive to circulating influenza variants and the emergence of unpredictable pandemic viruses than the egg-based lengthy traditional methods. This short timeline and high efficacy in mRNA vaccine technology are particularly advantageous during the outbreaks of new viruses or pandemics. Moderna and BioNTech companies aim to develop several mRNA vaccine candidates targeting pan-coronavirus, influenza, rabies, Zika, and HIV in the developmental pipeline [5,125]. Delivery of multiple mRNA vaccines will enable the induction of cross-immunity against multi antigens, targeting the variants and different pathogens. For example, Freyn et al. (2020) reported a multi-targeting mRNA influenza virus vaccine inducing broad protection in mice [123].

New mRNA vaccine innovations are expected to replace conventional vaccine platforms and develop more effective vaccines against recurrent and challenging pathogens or cancers in the near future. More development in delivery vehicles of mRNA will be required for safer, effective, and cold-chain-free mRNA vaccines. An example is the development of thermostable mRNA vaccines [106,132]. Further understanding of the mechanisms of action in the mRNA vaccines in vivo remains to be investigated, particularly in the area of studying the impact of innate immune responses by respective mRNAs, delivery systems, and specific target cells or organs to minimize the potential side effects.

Intramuscular injection is the common route of administration for currently licensed COVID-19 vaccines, inducing systemic humoral and cell-mediated immune responses and protection against lung infection, inflammation, severe disease, and death. Breakthrough infections can occur in COVID-19 vaccinees that can transmit SARS-CoV-2 to unvaccinated individuals. Intranasal dosing of ChAd-SARS-CoV-2-S adenoviral based vaccine was shown to induce sterilizing immunity preventing SARS-CoV-2 infection in both the upper and lower respiratory tracts in mice [145,146]. An intranasal mRNA nanoparticle vaccination was reported to induce anti-tumor immunity in mice, suggesting a possibility of intranasal mRNA vaccines [147]. The efficacy of intranasal vaccination is mostly demonstrated in mouse and other animal models. It remains questionable and to be determined whether the efficacy of intranasal vaccination will be reproducible in humans as observed in animal models. Additionally, intradermal vaccination would provide dose sparing effects, expanding the coverage under the situation of a COVID-19 vaccine shortage [148]. Future studies are needed on the intradermal or intranasal application of mRNA vaccines.

## Figures and Tables

**Table 2:** mRNA-based influenza vaccines.

Immunogens	mRNA Type	Model/Route/mRNA Dose (μg)	References
Hemagglutinin (HA) (H1N1, H3N2, H5N1 virus)	Codon optimized/protamine RNA	Mice: i.d./20 μg, 80 μg. Ferrets: i.m./20, 80, 250 ug Pigs: i.d./250 μg	[119]
HA (H1N1, A/Cal09)	Self-amplifying mRNA/Cationic nanoemulsion	Mice: i.m./0.1, 1,10 μg Ferrets: i.m./15, 45 μg	[120]
HA (H1N1pdm09)	Codon optimized/unmodified/LNPs	Mice: i.m./0.5, 5 μg NHPs: i.m./10 μg	[121]
HA (H10N8)	Nucleoside modified/LNPs	NHPs: i.m./50 μg mRNA +/− GLA (5 μg) adjuvant	[98]
HA (H1N1, A/Cal09)	Nucleoside modified (m1ψ-mRNA/LNPs)	Mice: i.d. (3, 10, 30 μg) Mice: i.m. (10, 30, 90 μg) Ferrets: i.m./30 μg	[122]
HA (H1, A/PR8)	Nucleoside modified (m1ψ-mRNA/LNPs)	Mice: i.d. (30 μg, single dose)	[97]
Mini-HA stalk, NA, M2, NP	Nucleoside modified (m1ψ-mRNA/LNPs)	Mice: i.d. (0.05, 0.5, 5, 10, 20 μg, single dose)	[123]
HA (H10N8, H7N9; H10 mRNA in phase 1)	Nucleoside modified/LNPs	Mice: i.d., i.m./10 μg, single dose; 0.4, 2 μg boost dose) Ferrets: i.d./10, 50, 200 μg NHPs: i.d., i.m./200, 400 μg Humans (phase 1): i.m./100 μg	[21]
HA (H10N8, H7N9; H10 mRNA, phase 1)	Nucleoside modified/LNPs	Adults: i.m./H7N9 mRNA 10, 25, 50 μg; i.m./H10N8 mRNA 25, 50, 75, 100, 400 μg; i.d./25, 50 μg	[22]

LNPs: lipid nanoparticles. NHPs: non-human primates. i.d.: intradermal, i.m.: intramuscular.

## Data Availability

Not applicable.
